# The Discrepant and Similar Responses of Genome-Wide Transcriptional Profiles between Drought and Cold Stresses in Cassava

**DOI:** 10.3390/ijms18122668

**Published:** 2017-12-12

**Authors:** Changying Zeng, Zehong Ding, Fang Zhou, Yufei Zhou, Ruiju Yang, Zi Yang, Wenquan Wang, Ming Peng

**Affiliations:** Institute of Tropical Bioscience and Biotechnology, Chinese Academy of Tropical Agricultural Sciences, Haikou 571101, China; zengchangying@itbb.org.cn (C.Z.); dingzehong@itbb.org.cn (Z.D.); fzhou_413@aliyun.com (F.Z.); zyf20080825@163.com (Y.Z.); wxxnyrj@126.com (R.Y.); yangzi8866@126.com (Z.Y.); wangwenquan@itbb.org.cn (W.W.)

**Keywords:** cassava, cold, drought, gene regulation, common response, specific response

## Abstract

**Background**: Cassava, an important tropical crop, has remarkable drought tolerance, but is very sensitive to cold. The growth, development, and root productivity of cassava are all adversely affected under cold and drought. **Methods**: To profile the transcriptional response to cold and drought stresses, cassava seedlings were respectively subjected to 0, 6, 24, and 48 h of cold stress and 0, 4, 6, and 10 days of drought stress. Their folded leaves, fully extended leaves, and roots were respectively investigated using RNA-seq. **Results**: Many genes specifically and commonly responsive to cold and drought were revealed: genes related to basic cellular metabolism, tetrapyrrole synthesis, and brassinosteroid metabolism exclusively responded to cold; genes related to abiotic stress and ethylene metabolism exclusively responded to drought; and genes related to cell wall, photosynthesis, and carbohydrate metabolism, DNA synthesis/chromatic structure, abscisic acid and salicylic acid metabolism, and calcium signaling commonly responded to both cold and drought. **Discussion**: Combined with cold- and/or drought-responsive transcription factors, the regulatory networks responding to cold and drought in cassava were constructed. All these findings will improve our understanding of the specific and common responses to cold and drought in cassava, and shed light on genetic improvement of cold and drought tolerance in cassava.

## 1. Introduction

Cold and drought are the most common and primary environments that adversely affect plant growth and crop production worldwide. Because plants are sessile and have to endure one or more environmental challenges, plants have developed multiple complex signaling networks involved in stress perception, signal transduction, and the expression of stress-related genes to cope with adverse environmental conditions during their long-term adaptation [1]. The calcium ion (Ca^2+^) is recognized as a secondary messenger and appears to play crucial roles in signal transduction under both cold and drought stresses, via the changes in intracellular Ca^2+^ concentration [2]. Several kinds of Ca^2+^ sensors, including calmodulin (CaM), CaM-like protein (CML), calcineurin B-like protein (CBL), and Ca^2+^-dependent protein kinase (CDPK), are induced in response to cold and drought [2]. Moreover, *Arabidopsis* transgenic plants with over-expression of *GmCaM4* or *VaCDPK20* exhibit enhanced stress tolerance [3,4]. Mitogen-activated protein kinase (MAPK), another secondary messenger, is an important mediator in signal transmission, connecting the perception of external stimuli to cellular responses. Previous studies have demonstrated that MAPKs are involved in cold, drought, or both cold and drought stresses [5,6]. Reactive oxygen species (ROS), such as superoxide, hydrogen peroxide, and hydroxyl radicals, are also secondary messengers and accumulate under cold and drought stresses. They are signals contributing to stress injury in plants, as transgenic plants over-expressing ROS scavengers or mutants with higher ROS-scavenging ability show increased tolerance to environmental stresses [7].

In addition to the secondary messengers, hormones have been demonstrated to be key players in the response to cold and drought. Of these, abscisic acid (ABA) plays a critical role in response to various stress signals [8]. Under osmotic conditions imposed by drought, the most rapid response of plants is closure of the stomata, mediated by ABA, to maintain their water balance. Accordingly, expression of several ABA biosynthesis genes, such as *NCED3* and *LOS6*/*ABA1*, are up-regulated by drought stress [9]. In addition to the ABA-dependent pathway, molecular analyses have revealed that ABA-independent pathways are also involved in transcriptional regulatory networks under drought stress [8,10]. However, under cold stress, the expression of genes is influenced largely through an ABA-independent pathway, because expression of some key genes (e.g., *ZEP* and *NCED*) involved in ABA biosynthesis is not obviously up-regulated by cold and there is little or no increase in ABA content in plants subjected to cold treatment [7,11]. It is noteworthy that the expression of *Arabidopsis* gene *RD29A* was subject to both ABA-dependent and -independent regulation under both drought and cold conditions [1]. In addition to ABA, hormones such as ethylene, salicylic acid (SA), and brassinosteroid (BR) are also involved in abiotic stress responses [12,13]. Moreover, these hormones interact with one another in regulating stress signaling and tolerance in plants [13].

Transcription factors (TFs) are also key regulators involved in plant abiotic stress signaling. Many cold-regulated genes (*COR*) have been identified as responding to cold treatment. The most characterized transcriptional cascade, with a large role in cold stress pathways, is called “ICE-CBF-COR” [1]. Of these, C-repeat binding factors (CBFs) are members of the AP2/EREBP family and are involved in regulation of ABA-independent expression of *COR* genes under cold stress. Constitutive- or over-expression of CBFs enhances cold stress tolerance in several plants [14,15]. Inducer of CBF expression (*ICE*), a member of the bHLH family, can directly interact with CBFs and regulate their expression upon cold treatment [16]. Moreover, the over-expression of *ICE*s in transgenic plants results in improved freezing tolerance [17]. ABA is a major player in response to drought stress, thus the TFs involved in ABA signaling pathways are related to drought stress. For example, ABRE-binding factor (ABF), of the bZIP family, can bind to the ABRE motif and activate ABA-dependent gene expression during drought stress [18]. Other important TFs, such as the MYC and myeloblastosis (MYB) proteins, are also known to function as activators in ABA-dependent regulatory pathways. Transgenic plants over-expressing both *AtMYC2* and *AtMYB2* showed an ABA-hypersensitive phenotype and improved osmotic stress tolerance [8,19]. In addition to the cold- or drought-specific induced TFs, some others (e.g., *WRKY45*, *ZAT12*, and *NAC6*) are involved in both cold and drought stresses.

To further increase the understanding of signaling pathways for the improvement of crop stress tolerance, it is essential to know how plants respond to stresses and which genes and pathways are involved in the stress tolerance. Over recent decades, significant progress has been made regarding our knowledge of plant response to abiotic stresses, and thousands of genes and tens of signaling pathways have been identified concerning cold and drought [8,20]. Moreover, many of these genes were found to be regulated commonly or specifically in response to drought and cold stresses in several plants, including *Arabidopsis* [21], rice [22], and cotton [23] at the transcriptional level. However, in tropical plants (e.g., cassava), similar studies are very limited, and the molecular mechanisms underlying tolerance to both drought and cold stresses remain largely unknown.

Cassava (*Manihot esculenta* Crantz) is an important cash crop and provides staple food for over 750 million people in tropical and sub-tropical areas [24]. It is a major crop for starch, bio-fuel production, and animal feed, owing to its starch-enriched root [25]. Cassava is generally thought to be drought-tolerant, however, similarly to other crops, this drought tolerance is usually at the cost of restrained growth and decreased economic yield [25]. As a tropical root crop, cassava is native to a warm habitat and is very sensitive to cold, which greatly damages the plants and reduces yield [26]. Thus, it is very important to increase our understanding of the molecular mechanisms of cold and drought stresses in cassava. With rapid development of next generation sequencing techniques and the release of the cassava draft genome [27,28], much progress has been made in large-scale identification of genes involved in cold [26,29] and drought [24,30,31] responses, respectively, in cassava at the transcriptional or proteomic levels. However, very few studies have focused on comparisons of transcriptome profiles in response to cold and drought stresses in cassava, and the related signaling pathways have not been adequately explored. In this study, the same cassava cultivar leaves (including folded and full-expanded leaves) and roots were respectively collected at 0, 6, 24, and 48 h after cold treatment and 0, 4, 6, and 10 days after drought treatment, to investigate the genome-wide transcriptome changes through RNA-seq technology. The results will provide new insights into the signaling regulation networks responding to cold and drought in cassava, and will also provide solid bases for cassava improvement in regard to these two abiotic stresses.

## 2. Results

### 2.1. Stress Responses and Transcriptional Profiling of Cassava

Compared with controls, there were respective distinct symptoms in the cold- and drought-treated plants as the treatment time progressed (Figure S1). For the cold treatment, the plants were badly wilted at 6 h, their top leaves were curved at 24 h, and symptoms were more serious at 48 h. The drought-treated plants were badly wilted at 4 days, their lower leaves began to fall off at 6 days, and only a few upper leaves remained at 10 days (Figure S1).

To investigate the spatial and temporal gene expression in response to abiotic stresses in cassava at the transcriptional level, different tissues, including folded leaves (FL, the first three folded leaves), fully expanded leaves (FEL, three most newly fully expanded leaves), and fibrous roots (RT), were respectively collected at 0, 6, 24, and 48 h after cold treatment and 0, 4, 6, and 10 days after drought treatment. Overall, 88.2 million raw reads of 35 bp in length were obtained by single end sequencing with the Illumina GAII platform. After sequence pre-processing, 83.4 million adapter-trimmed clean reads were kept, and 95.8% of them were mapped to the cassava reference genome (Available online: http://www.phytozome.net) with a maximum of two mismatches.

Overall, 18,271 genes, approximately two-thirds of the annotated genes in the reference genome, were expressed across drought-treated samples. Comparatively, a similar number of genes (17,352) were expressed across cold-treated samples. Interestingly, in either cold or drought treatment, the numbers of expressed genes were quite similar in the samples of all examined time points within the same tissue, as well as in different tissues when considering all the time points together (i.e., a gene was counted if it was expressed in at least one of four time points within the same tissue) (Figure S2). In addition, in both cold and drought treatments, most of these genes were commonly expressed while only a few were exclusively expressed in FL, FEL, and RT, respectively (Figure S2). The results indicated that there were similar amounts of expressed genes in different tissues (e.g., FL, FEL, and RT) of cassava to maintain a kind of housekeeping effect during cassava growth and development, regardless of experiencing abiotic stress or not. Once subjected to abiotic stress, the expression of these genes in cassava would be up- or down-regulated to cope with the adverse environments.

In addition, seven genes of interest were selected and their expression patterns were validated with high correlation coefficient (r = 0.94) between RNA-seq and qRT-PCR methods (Table S1).

### 2.2. Transcriptome Changes in Response to Drought and Cold

**Figure 1:**
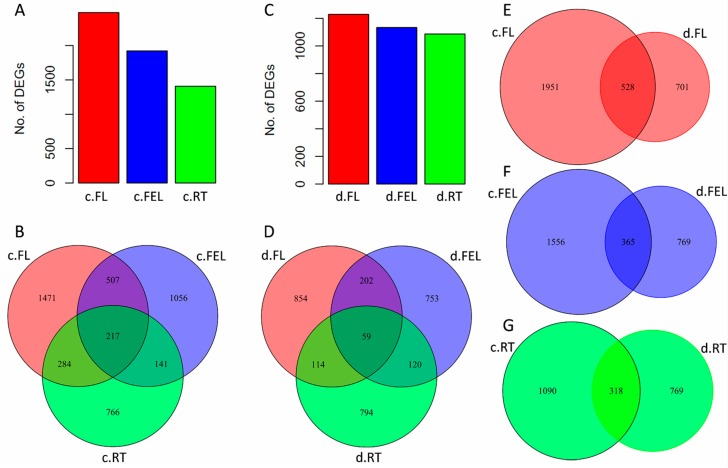
Transcriptome profiling of cassava in response to cold and drought stresses. Differentially expressed (DE) genes identified in FL, FEL, and RT under cold stress (**A**) and their Venn diagrams (**B**). DE genes identified in FL, FEL, and RT under drought stress (**C**) and their Venn diagrams (**D**). Venn diagrams of DE genes between cold and drought stresses in FL (**E**), FEL (**F**), and RT (**G**), respectively. c.FL, c.FEL, and c.RT represent FL, FEL, and RT samples under cold stress, while d.FL, d.FEL, and d.RT represent FL, FEL and RT samples under drought stress.

To reveal the transcriptome changes affected by abiotic stresses, differentially expressed (DE) genes were identified under cold and drought, respectively. Under cold treatment (signified by prefix “c.”), there were 2479, 1921, and 1408 DE genes in c.FL, c.FEL, and c.RT, respectively, with an increased gradient change of DE gene number from bottom to top of a plant (Figure 1A). Unlike expressed genes, most of these DE genes were exclusively identified in c.FL (1471), c.FEL (1056), and c.RT (766), but only 217 were commonly identified in all of these tissues (Figure 1B). Under drought treatment (signified by prefix “d.”), a similar gradient change of DE gene number was observed: d.FL (1229) > d.FEL (1134) > d.RT (1087), although the numbers were slightly lower compared with those of the cold treatment (Figure 1C). Similarly, most of these DE genes were also exclusively identified in d.FL (854), d.FEL (753), and d.RT (794), and only a few (59) were commonly identified (Figure 1D). Thus, in response to cold and drought, expression of DE genes was more influenced in cassava leaves than in roots, and the majority exhibited tissue-specific expression patterns while only a small proportion were commonly influenced across tissue types.

The DE genes were compared between cold and drought treatments within the same tissue to explore their roles and expression responses to different abiotic stresses. In FL, 1951 (61.4%) DE genes were uniquely identified under cold treatment (Figure 1E). Functional category enrichment showed that these genes were significantly enriched in calcium signaling and ABC transporters (Figure S3). As compared, 701 (22.0%) DE genes were uniquely identified under drought and 528 (16.6%) were identified under both cold and drought (Figure 1E). These two groups of genes were commonly enriched in several categories, including cell wall, photosynthesis, and abiotic stress (Figure S3). In FEL, 1556 (57.8%) and 769 (28.6%) DE genes were uniquely identified in cold and drought, and they were enriched in photosynthesis and tetrapyrrole synthesis, and redox and abiotic stress, respectively (Figure S3). In total, 365 (13.6%) DE genes were commonly found in response to cold and drought (Figure 1F), however, no significantly enriched categories were observed. In RT, 1090 (50.0%) DE genes were uniquely identified under cold treatment (Figure 1G), but there were no significantly enriched categories. A total of 769 (35.3%) DE genes were uniquely identified under drought, and 318 (14.6%) were commonly identified under both cold and drought (Figure 1G); these genes were significantly enriched in secondary metabolism and abiotic stress, respectively (Figure S3). These results also revealed that the expression of genes was more influenced by cold (~55%) than drought (~30%), and only a few of genes (~15%) were commonly influenced by both cold and drought in FL, FEL, or RT (Figure 1E–G).

To further characterize the functions of DE genes that were uniquely or commonly identified in cold and drought stress, fold-change-based clustering analysis and functional category enrichment were performed for each group, respectively.

### 2.3. DE Genes Specifically Responding to Cold

**Figure 2:**
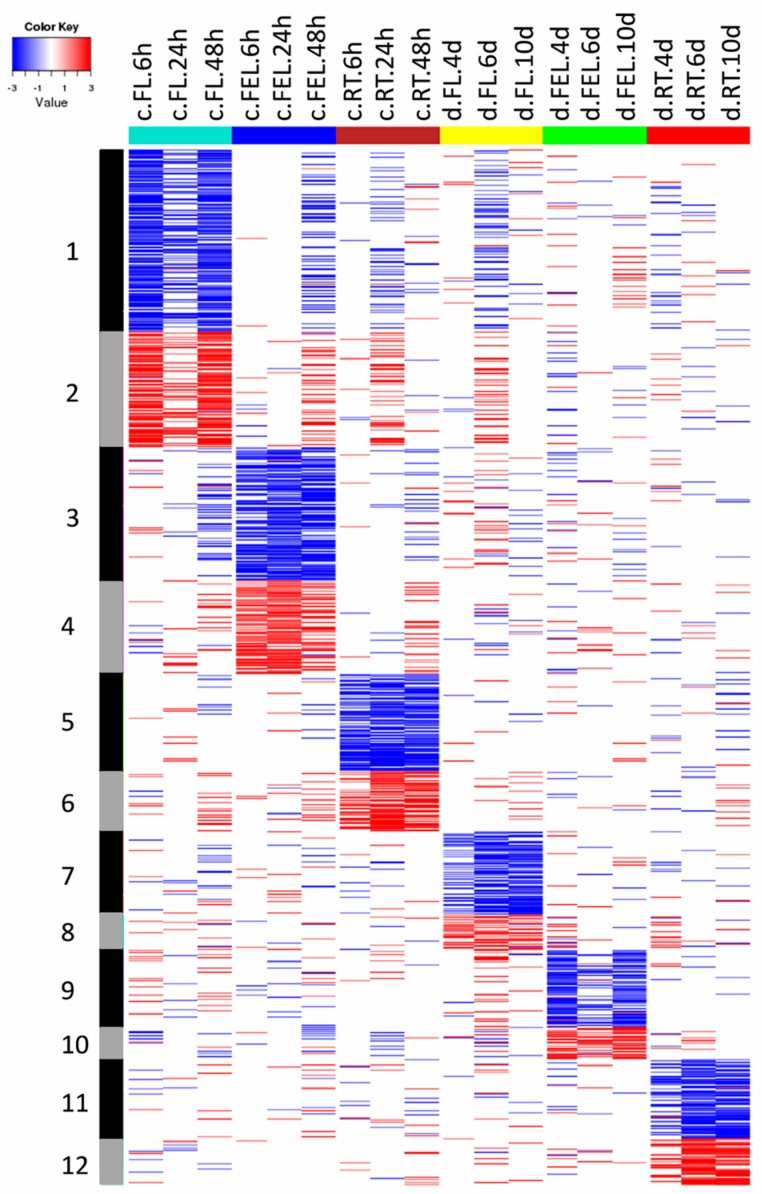
Heatmap of DE genes significantly changed in one tissue responding to cold or drought stress. In total, 12 groups of genes were identified. Blue represents down-regulation, while red represents up-regulation. Samples were named by combination of stresses (“c” for cold, and “d” for drought), tissues, and time points. For examples, c.FL.6h represents sample from FL under cold treatment for 6 h, while d.FL.4d represents sample from FL under drought treatment for 4 days.

**Figure 3:**
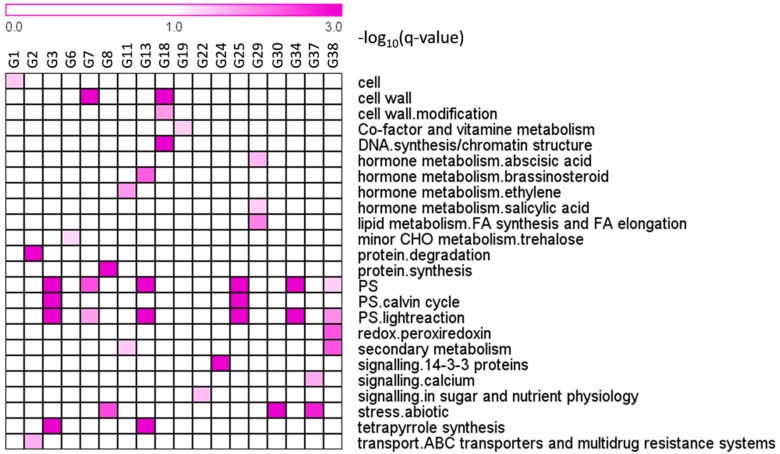
Functional category enrichment. Groups were indicated at the columns, while functional categories derived from MapMan annotation were indicated at the rows.

In total, 38 groups (G1–G38) comprising 5030 DE genes that were differentially expressed in at least one tissue, were identified (Table S2). As shown in Figure 2, the expression of genes from groups G1–G6 was significantly changed in only one tissue in response to cold. For example, the expression of 604 genes in G1 and 383 genes in G2 was significantly decreased and increased in FL, respectively. Category enrichment showed that these genes were mainly involved in cell-related metabolism, and protein degradation and ABC transporters, respectively (Figure 3). There were 439 genes in G3 and 311 in G4 of which the expression was significantly decreased and increased in FEL, respectively. The enriched categories in G3 included Calvin cycle and light reaction of photosynthesis, and tetrapyrrole synthesis, however, no enriched categories were found in G4 (Figure 3). Likewise, there were 319 genes in G5 and 201 in G6 whose expression was significantly decreased and increased in RT, respectively (Figure 2). No enriched categories were found in G5, however, the genes of G6 were mainly involved in trehalose metabolism (Figure 3).

**Figure 4:**
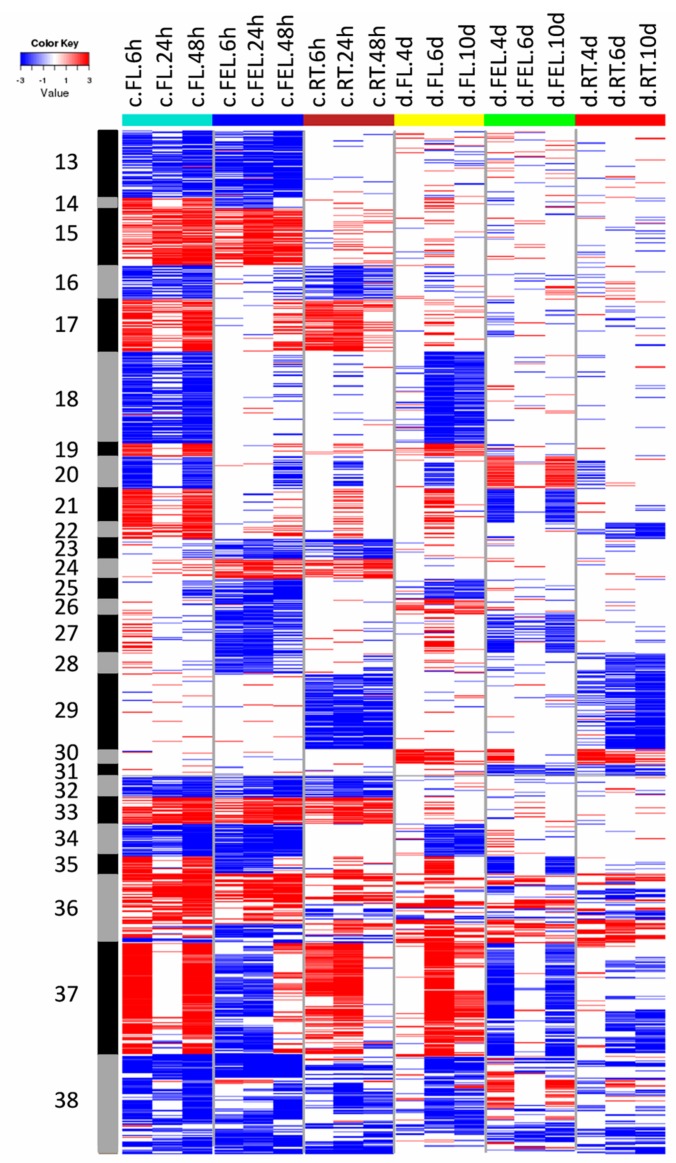
Heatmap of DE genes significantly changed in at least two tissues responding to cold and/or drought stresses. In total, 26 groups of genes were identified. Blue represents down-regulation, and red represents up-regulation. Samples were named by combination of stresses (“c” for cold, and “d” for drought), tissues, and time points as indicated in Figure 2.

The expression of genes from groups G13–G17 was significantly changed in more than one tissue exclusively in response to cold (Figure 4). For example, the expression of genes in G13 was significantly decreased in both FL and FEL, and these genes mainly participated in hormone metabolism (e.g., BR), light reaction of photosynthesis, and tetrapyrrole synthesis (Figure 3). On the contrary, the expression of G15 genes increased in both FL and FEL. Similarly, genes decreased in G16 but increased in G17 in both FL and RT, genes decreased in G23 but increased in G24 in both FEL and RT, and genes decreased but increased in all the three tissues, respectively (Figure 4). However, no significantly enriched categories were found in most of these groups (Figure 3).

Together, the results indicated that DE genes, which exclusively responded to cold, were mainly involved in cell related metabolism, protein degradation, Calvin cycle and light reaction of photosynthesis, tetrapyrrole synthesis, and BR metabolism.

### 2.4. DE Genes Specifically Responding to Drought

The expression of genes from groups G7–G12 was significantly changed in only one tissue in response to drought (Figure 2). The expression of genes of G7 and G8 was significantly decreased and increased in FL, respectively. The enriched categories in G7 included cell wall and light reaction of photosynthesis, and those in G8 included protein synthesis and abiotic stress (Figure 3), which was quite different to those of cold responses in the same tissue. Similarly, the gene expression of G9 and G10 was significantly decreased and increased in FEL, respectively. No enriched categories were found in these two groups (Figure 3). The expression of 259 genes in G11 and 156 in G12 was significantly decreased and increased in RT, respectively (Figure 2). The genes in G11 were enriched in ethylene metabolism and secondary metabolism, but no enriched categories were found in G12 (Figure 3).

In addition to genes that were differentially expressed in only one tissue, many genes whose expression was significantly changed in more than one tissue were also observed exclusively in response to drought. For example, the expression of genes in G30 was significantly increased in FL and RT under drought, and these genes mainly participated in abiotic stress (Figure 3 and Figure 4). The expression of genes in G31 was significantly decreased in FEL and RT under drought, but no enriched categories were observed (Figure 3 and Figure 4).

Together, the results indicated that DE genes, which exclusively responded to drought, were mainly involved in cell wall, protein synthesis, light reaction of photosynthesis, abiotic stress, and ethylene metabolism.

### 2.5. DE Genes Responding to Both Cold and Drought

Many genes that showed significantly changed expression in both cold and drought were also observed. The expression of genes in G18 and G19 was significantly decreased and increased, respectively, in FL under both cold and drought (Figure 4). Category enrichment showed that these genes were involved in cell wall and DNA synthesis/chromatic structure, and co-factor and vitamin metabolism, respectively (Figure 3). In addition, groups of genes whose expression was changed under both cold and drought stresses were also observed in FEL (e.g., G27) and RT (e.g., G29) (Figure 4). The enriched categories of G29 included hormone metabolism of ABA and SA, and FA synthesis and FA elongation, but no enriched categories were observed in G27 (Figure 3). Interestingly, the expression of photosynthesis-related genes was significantly decreased in response to both cold and drought, although there were different expression patterns of these genes (e.g., G25, G34, and G38) (Figure 4). In addition, the expression of genes involved in Ca^2+^ signaling and abiotic stress (e.g., G37) was also significantly changed in response to both cold and drought.

In the following subsections, a few special and interesting pathways were further inspected to illustrate their respective responses to cold and drought stresses.

### 2.6. Photosynthesis and Carbohydrate Metabolism

Genes related to photosynthesis, including both light reaction and Calvin cycle, were dramatically influenced by either cold or drought, or both of these stresses (Figure 3). It is noteworthy that the expression of all photosystem I and II DE genes was significantly decreased in leaves (Table S2). Consistently, DE genes related to electron carrier (e.g., ferredoxin-NADP^+^ oxidoreductase 2), cyclic electron flow-chlororespiration, and ATP synthase of light reaction were also decreased (Table S2), although they exhibited stress-specific response patterns (e.g., cyclic electron flow-chlororespiration for cold and ATP synthase for drought). Likely, the expression of Calvin-cycle-related genes, such as three rubisco small subunit, four glyceraldehyde 3-phosphate dehydrogenase (two *GAPA* and two *GAPB*), two fructose-1,6-bisphosphatase (*FBPase*), two sedoheptulose bisphosphatase (*SBPase*), and two phosphoribulokinase (*PRK*), was significantly decreased in response to cold and drought (Table S2). In contrast to light reaction and Calvin cycle, the expression of only a few genes related to photorespiration was changed (Table S2).

Tetrapyrroles play vital roles in photosynthesis and respiration. There are three branches, including the chlorophyll branch, siroheme branch, and heme branch, in the tetrapyrrole biosynthesis pathway in plants. However, only the expression of genes located in the chlorophyll branch was significantly influenced, especially in FL and FEL in response to cold, in which the expression of magnesium chelatase and protochlorophyllide reductase was dramatically decreased (Table S2). These results suggested that photosynthesis metabolism was greatly influenced by tetrapyrrole biosynthesis through the chlorophyll branch, specifically in response to cold.

Starch and sucrose metabolisms are closely linked to photosynthesis. As expected, the expression of starch and sucrose metabolism genes was greatly depressed by cold and drought. For example, the expression of ADP-glucose pyrophosphorylase (*AGPase*) and starch synthase (*SS*) in starch biosynthesis [32], as well as alpha amylase, beta amylase, and glycosyl transferase in the starch degradation pathway [32] was significantly decreased in response to cold and drought (Table S2). Similar changes were observed in sucrose-degradation-related genes such as fructokinase, hexokinase, sucrose synthase (*SUS*), and sucrose transporters (*SUT*) (Table S2).

Together, the results indicated that cold and drought dramatically depressed the expression of photosynthesis genes mainly through reduced accumulation of chlorophyll and then inhibited the processes of carbohydrate metabolism.

### 2.7. Response of Abiotic-Stress-Related Genes

In total, 78 genes related to abiotic stress were identified in response to either cold or drought after manual curation. The most dominant category was related to heat stress (53 genes), followed by drought/salt (18 genes), cold stress (five genes), and touch/wounding (two genes, Table S3).

Hierarchical clustering analysis grouped these genes into four main clusters (Figure S4). Overall, the expression of genes in cluster A1 (14%, 11 genes) was significantly decreased under both stresses (except for c.FL), and the expression of genes in cluster A3 (33%, 26 genes) was dramatically increased, especially in the early response to drought. *ERD4*, which encoded an early-responsive to dehydration stress protein [33], was included in cluster A1, while most genes in cluster A3 were related to heat stress (Table S3). In comparison, the expression of genes in cluster A2 (32%, 25 genes) was significantly decreased under cold but increased under drought stress, whereas cluster A4 (21%, 16 genes) showed opposite trends, except for those induced in FL under drought stress. As expected, several dehydration-responsive genes, including *ERD3*, *QUL2*, and *DI19* [34,35], were included in these two clusters. These results suggested that abiotic-stress-related genes had different responses to cold and drought.

### 2.8. Response of Genes Related to Ca^2+^ Signaling, MAPK, and ROS

Ca^2+^ sensors, MAPKs, and ROS-scavenging enzymes are important secondary messengers involved in abiotic stresses [2,5,7], thus it is of great interest to determine their expression changes in response to cold and drought in cassava. In total, 13 Ca^2+^ sensor-related genes, including three *CaM*s, six *CML*s, one *CBL*, and three *CDPK*s, were identified (Table S4). Four of them were responsive to both cold and drought, while the remaining nine were exclusively responsive to cold. Interestingly, most of the genes that responded to cold were up-regulated, whereas those responsive to drought were down-regulated (Table S4). A total of 13 *MAPKs*, including six *MAPKs* and seven *MAPKKK* members, were identified. Similar to Ca^2+^ sensors, most *MAPKs* were differentially expressed in response to cold, or both cold and drought, but very few exclusively responded to drought (Table S4). In total, 10 ROS-scavenging enzymes, including two superoxide dismutase (*SOD*), two catalase (*CAT*), four ascorbate peroxidase (*APX*), and two glutathione reductase (*GR*), were identified. Unlike Ca^2+^ sensors and *MAPKs*, most ROS-scavenging enzymes exclusively responded to cold or drought, and only one responded to both of these stresses (Table S4). Together, these results implied that Ca^2+^ sensors, MAPKs, and ROS-scavenging enzymes played important roles in cassava abiotic stress responses, but with different emphases for cold and drought.

### 2.9. Roles of Genes Related to Hormone Biosynthesis

Hormones usually play important roles in plant developmental processes and adaptation to changing environments. In this study, a total of 111 hormone-related genes, referring to ABA, auxin, BR, ethylene, gibberellic acid (GA), jasmonic acid (JA), SA, and cytokinin (CK), were differentially expressed in response to cold and drought. The top four hormones with most abundant genes were ethylene (35 genes), auxin (27), ABA (13), and GA (13), followed by BR (11) and SA (7) (Table S5).

To reveal the role of hormones responding to cold and drought, the expression of genes related to biosynthesis of each hormone was examined, respectively. Nine-*cis*-epoxycarotenoid dioxygenase (NCED) and ABA-aldehyde oxidase (AAO) are two key enzymes in the ABA biosynthetic pathway [9]. The expression of *NCED4* and *NCED5* was significantly decreased in FL and FEL under cold stress, while the expression of *NCED7*, *NCED8*, and *AAO2* was significantly decreased in RT under both cold and drought. Besides this, the expression of *AAO4* was greatly induced in FEL exclusively in response to cold (Table S5).

In the auxin biosynthetic pathway, the expression of *ILL6* (IAA-leucine resistant like gene 6), which encodes IAA-amino acid conjugate hydrolase involved in generating IAA from the hydrolysis of IAA-amino acid conjugates, was significantly depressed in RT under cold stress (Table S5). Consistently, *PIN1*, which encodes an auxin efflux carrier, showed a very similar expression trend. In addition, the expression of *PIN3* was also decreased, although it preferentially responded to cold in FL and FEL.

The expression of three genes *DWF1*, *DWF5*, and *DWF7*, which are involved in different steps of the BR biosynthesis pathway [36], was dramatically decreased in FL and FEL under cold stress (Table S5).

ACC synthetase (ACS) and ACC oxidase (ACO) are two key enzymes that catalyze the final two steps of ethylene biosynthesis [37]. Although the expression of *ACS8*, *ACO4*, and *ACO5* was greatly depressed, the former two responded to both cold and drought in FL and FEL, whereas *ACO5* preferentially responded to cold in RT.

The expression of GA biosynthetic genes, including copalyl diphosphate synthase (*CPS*) and *GA20OX1*, was greatly induced in response to cold and drought. In contrast, the expression of JA biosynthetic genes, including lipoxygenase (*LOX*), allene oxide synthase (AOS), and oxophytodienoate reductase (*OPR*), was all significantly depressed, although *LOX2* and *OPR3* preferentially responded to drought, and *LOX6* and *AOS1* responded to both cold and drought.

Together, these results suggested diverse roles of hormone genes in response to cold and drought stresses.

### 2.10. Roles of Transcription Factor (TF)

The expression changes of TF responding to cold and drought were well resolved in our RNA-seq data. In total, 1810 TF genes were expressed in at least one sample, and 270 (14.9%, representing 24 families) of them were differentially expressed in response to cold and drought stresses (Table S6). The three most abundant TF families were MYB (29 genes), bHLH (26), and AP2/EREBP (25), followed by HB (23), C2H2 (23), WRKY (17), and bZIP (16). Of the 270 differentially expressed TFs, most (57.0%) of them were exclusively in response to cold, whereas 25.6% were specifically identified in drought, and about 17.4% were identified in both cold and drought (Table S6).

**Figure 5:**
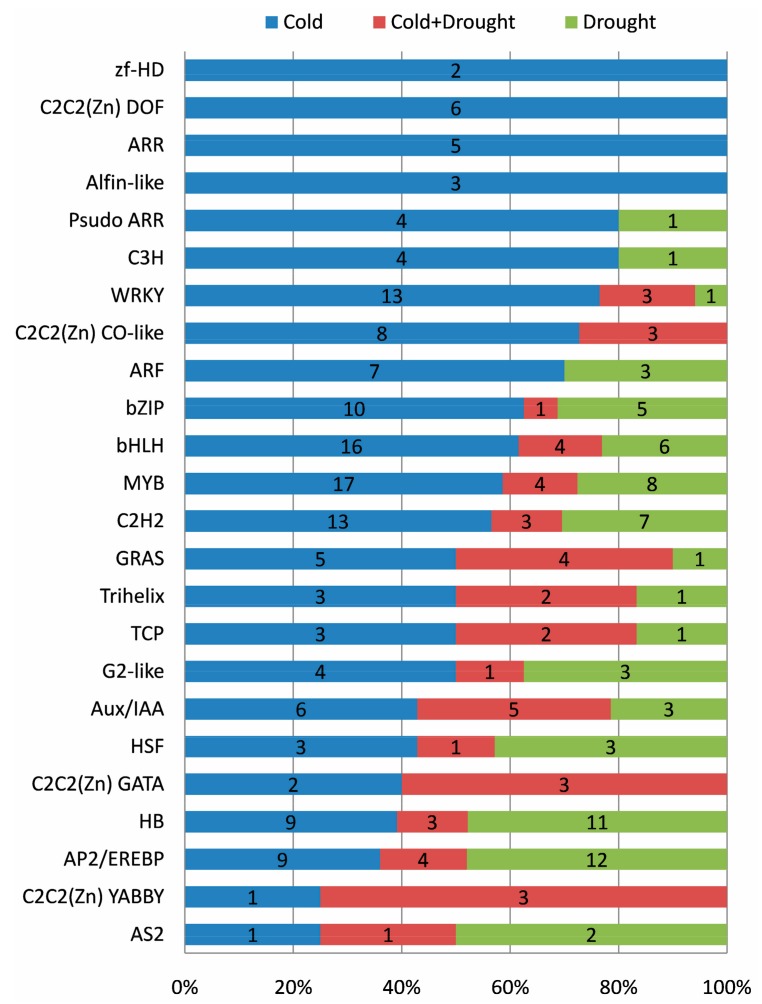
Distribution of transcription factor families differentially expressed in response to cold, drought, or both cold and drought stresses.

Stress-specific expression trends were also inspected in each TF family, respectively. All members of zf-HD, C2C2 (Zn) DOF, ARR, and Alfin-like families were identified to exclusively respond to cold (Figure 5), of which, *ARR1* and *ARR12* were involved in low temperature mediated inhibition of root growth by reducing auxin accumulation. Moreover, four out of five DE genes from the ARR family were involved in CK signaling (Table S6), suggesting that CK played major roles in response to cold stress in cassava.

Most members from Psudo ARR, C3H, WRKY, C2C2(Zn) CO-like, ARF, bZIP, bHLH, and MYB families preferentially responded to cold (Figure 5), of which, *PRR7* from Psudo ARR family was an essential component contributing to a temperature-sensitive circadian system; *WRKY39* functioned as a positive regulator in response to heat stress through SA- and JA-activated signaling pathways; *GBF3* from the bZIP family was involved in both cold and drought responses; *AIF4*, *bHLH1*, and *ICE1* from the bHLH family were involved in cold or freezing tolerance response; and *RVE1* and *MYB14* from the MYB family participated in the regulation of cold tolerance or cold acclimation (Table S6). In addition, multiple TF families involved in hormones were also identified, e.g., C3H family (*AtTZF2* and *AtTZF3*), WRKY family (*WRKY33* and *WRKY40*), bZIP family (*GBF3* and *ABI5*), and bHLH family (*JAM2*, *AKS2*, and *AKS3*) for ABA; WRKY family (*WRKY33*) for ethylene; WRKY family (*WRKY51* and *WRKY70*) and bHLH family (*JAM2*) for JA; and WRKY family (*WRKY4* and *WRKY70*) for SA (Table S6), suggesting that these hormones played major roles in response to cold stress.

In comparison, majority of the members of the HB, AP2/EREBP, and AS2 families preferentially responded to drought stress, whereas most members of the C2C2 (Zn) GATA and C2C2 (Zn) YABBY families responded to both cold and drought (Figure 5). Accordingly, we found that *HB7* and *HB12* from the HB family participated in drought response via an ABA-dependent manner, and *SHN1*, *RAP2.4*, *ANT*, *CBF4*, and *ABR1* from the AP2/EREBP family were involved in drought response through hormones such as ethylene, ABA, and auxin. Notably, an AP2/EREBP member (*CBF3*) previously reported to be involved in low temperature and ABA response, was specifically differentially expressed in RT under drought stress in our study (Table S6).

Overall, the results revealed that TF members (even within the same family) exhibited exclusive and common expression responses to cold and drought, which might be regulated through hormone signaling.

## 3. Discussion

### 3.1. Specific and Common Responses to Cold and Drought

To adapt to unfavorable environmental conditions caused by the clearly differing cold and drought stresses, the expression of numerous genes was specifically changed [7]. Interestingly, the majority of these genes exhibited tissue-specific expression (Figure 2), reflecting that there are subsets of genes responsible for certain roles in different tissues responding to either cold or drought. For example, in FL, the expression of genes from G1, involved in cell-related metabolism, was significantly lower in response to cold, whereas genes from G7 were related to cell wall and their expression was significantly decreased under drought condition (Figure 2 and Figure 3). These results indicated that, although both cold and drought diminished plant growth, the responses may have a different emphasis, e.g., cell activities such as cell division for cold and cell wall metabolism for drought [38,39]. Similarly, in FEL and RT, tissue-specific expression patterns were also observed in response to only cold or drought stress, respectively (Figure 2).

Some genes whose expression was significantly changed in at least two tissues also specifically responded to cold or drought stress. For example, genes from G13 were significantly decreased in both FL and FEL under cold conditions, and they were enriched in BR metabolism, tetrapyrrole synthesis, and light reaction of photosynthesis (Figure 3), of which, *STE1* encoded a BR biosynthetic enzyme [40]; *HEMB1* and *CLH1* respectively participated in chlorophyll biosynthesis and degradation [41,42], which is one branch of the tetrapyrrole synthesis pathway; and many genes involved in the light reaction of photosynthesis, e.g., *PSAD1*, *PSAE2*, *PSAO*, *PSBW*, *PSBP1*, *LHCA1*, and *LHCA2*, were also observed (Table S1). In contrast, genes from G30 were significantly up-regulated in FL and RT under drought (Figure 3). They mainly participated in response to abiotic stress. Moreover, six out of eight genes related to abiotic stress were heat shock proteins, supporting that heat shock proteins play important roles in drought stress in cassava [30].

Apart from genes that specifically responded to cold or drought, some common sets of genes were also identified (Figure 3), since cold and drought may activate some common reactions in plants [7]. More specifically, cold and drought could trigger similar primary effects at the cellular level and in downstream signal transduction chains [43]. For example, genes from G25, G34, and G38 were greatly depressed in leaves in response to both cold and drought (Figure 4), and they were enriched in photosynthesis metabolism, confirming suppression of photosynthesis-related genes under both cold and drought [30,31,44]. In addition, the expression of genes from G37 was also significantly changed in response to cold and drought. These genes were involved in abiotic stress as well as Ca^2+^ signaling, which has been well demonstrated to participate in signal transduction under many abiotic stresses such as cold, drought, and salt [1,7]. Tissue-specific expression patterns was also observed for the genes commonly responding to cold and drought. The expression of genes from G29 was greatly repressed exclusively in RT under these two stress conditions (Figure 4), and these genes were involved in ABA and SA metabolism, which are the key signaling hormones for responses to multiple abiotic stresses [10,45,46].

### 3.2. Regulatory Networks Involved in Cassava Response to Cold and Drought

**Figure 6:**
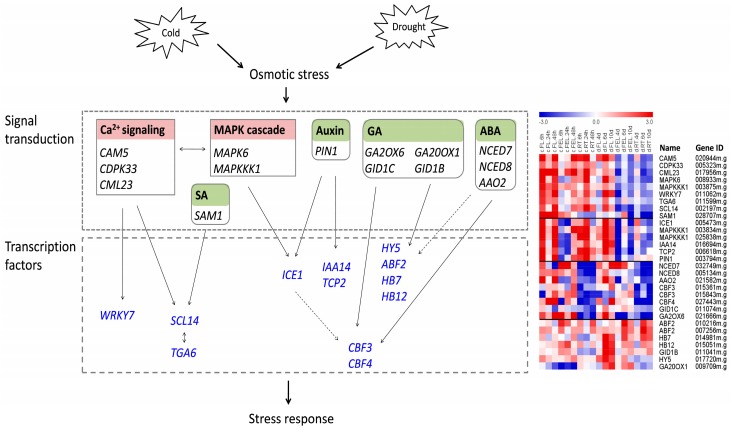
A model of transcriptional regulatory networks involved in cold and drought stresses in cassava. DE genes were mapped to the regulatory networks based on Mapman annotation, gene co-expression patterns, or both. Solid lines indicate the interactions that were in accordance with previous reported studies, while dash lines represent the interactions reported in other plants but not confirmed in our study. Transcription factors were marked by blue. Heatmaps of all genes in the networks were shown on the right.

In recent decades, thousands of genes and tens of metabolic and signaling pathways have been identified during cold and drought stresses [8], of which, genes related to Ca^2+^ sensors, MAPKs, ROS, and hormones are considered as important secondary messengers involved in signal transduction, and TF genes are recognized as key players involved in transcription regulation [1,8,45]. However, few such studies have been conducted in tropical plants like cassava. To reveal the common and different characteristics of cold and drought response mechanisms between cassava and other plants, DE genes were mapped to the related regulatory networks based on MapMan annotations, gene co-expression pattern, or both (Figure 6).

The Ca^2+^ sensors (including CaM, CML, CBL, and CDPK) play important roles in cold and drought stresses. In our study, 13 differentially expressed Ca^2+^ sensors were identified, of which, *CAM5*, *CML23*, and *CDPK33* were co-expressed and greatly induced in all examined tissues in response to cold, as well as in FL in response to drought (Figure 6). In *Arabidopsis*, *CAM5* and *CDPK33* were involved in stomatal closure [47,48], indicating that their homologues in cassava might play similar roles under stress conditions. *CML* genes were reported to interact with diverse TFs (e.g., *WRKY53* and *TGA3*) in plant defense responses [49]. As expected, one bZIP (*TGA6*), which was functionally redundant with *TGA2* [50], and one WRKY (*WRKY7*) TF was co-expressed with *CML23*, together with one SA-related gene (*SAM1*) and one GRAS TF (*SCL14*), which interacted with *TGA2* and affected the transcription of stress-responsive genes [51]. *TGA2* and *TGA6* were involved in the activation of SA-responsive genes; moreover, application of SA resulted in reduced stomatal aperture and conductance [52]. In addition, two MAPKs, *MAPK6* and *MAPKKK1*, which were involved in temperature and osmotic stresses [5], were also co-expressed with these genes. Together, these results indicated a Ca^2+^ signaling, MAPK, and SA hormone-mediated sub-network involving stomatal movement under cold and drought stresses in cassava.

*ICE1*, a MYC-type bHLH transcription factor, is the most upstream TF in the cold signaling pathway [16]. As expected, *ICE1* was significantly induced in response to cold stress, although its expression was depressed in FEL during the early stage of cold treatment. Besides, *ICE1* was also induced in FL but repressed in FEL and RT under drought condition (Figure 6). Two auxin-related genes, including an auxin efflux carrier *PIN1* and an auxin inducable Aux/IAA type TF *IAA14* [53], were co-expressed with *ICE1* (Figure 6), indicating that auxin and *ICE1* might interact pathways in cold and drought stresses in cassava. Another two genes, *MAPKKK1* and *TCP2*, were also co-expressed with *ICE1*. Numerous studies have demonstrated that *ICE* genes can directly interact with CBFs and regulate the expression of CBFs responding to cold stress [1,16]. Although three CBFs, including two *CBF3* and one *CBF4*, were differentially expressed under stress conditions, their expression trends were differed from that of *ICE1*, supporting that the CBF regulon in cassava may differ from its counterparts in *Arabidopsis* [26,29], or *ICE1* may regulate the expression of CBFs by controlling the stability of ICE1 protein as recently proposed [54].

ABA is a major hormone with important roles in plants osmotic stresses. NCED and AAO are two key enzymes in the ABA biosynthetic pathway. Here, three genes (*NCED7*, *NCED8*, and *AAO2*) were uniformly depressed in RT under both cold and drought, although they showed some increase in leaves (Figure 6). *CBF* genes are mainly induced by cold stress, but, to some extent, they can also be mediated by drought through an ABA-dependent pathway [1,10]. Moreover, CBFs are necessary for regulation of genes (e.g., *GA2OX6*) related to GA metabolism pathways [55]. Consistent with previous reports in other species, we observed that three ABA biosynthesis genes (*NCED7*, *NCED8*, and *AAO2*), three CBFs (two *CBF3* and one *CBF4*), together with two GA metabolism genes (*GID1C* and *GA2OX6*) were co-expressed (Figure 6), suggesting that this sub-network functioned in cold and drought stresses in cassava, especially in root tissue. Some exceptions were also observed. ABFs are bZIP TFs that can bind to the ABRE motif and regulate ABA-dependent gene expression [18]. However, the expression patterns of two *ABF2* genes were quite different to those of the three ABA biosynthesis genes (Figure 6). Similar results were observed for one bZIP TF (*HY5*) and two HB TFs (*HB7* and *HB12*), although they were ABA-dependent and responded to drought stress [56], as well as for one GA synthesis enzyme *GA20OX1* and one GA receptor *GID1B* (Figure 6). These results strongly suggested that *HB7* and *HB12* and the related co-expressed TFs and hormone genes constituted a sub-network and played important roles in cold and drought stresses in cassava via an ABA-independent pathway, which was in agreement with our recent independent study [30]. Taken together, these results provide novel insights into the regulatory networks of cold and drought stresses in cassava.

### 3.3. Comparison of Different Omics Studies Related to Cold or Drought Stress in Cassava

To date, only a few studies were reported concerning cold [26,29] or drought [24,30,31] stress in cassava. Comparing our work with these studies, two major differences were found: (1) Different types of tissues were used, e.g., most previous studies used pooled samples, while our study used separate FL, FEL, and RT to profile the cold- and drought-responsive transcriptional variations, and revealed that the majority of DE genes exhibited tissue-specific expression patterns and only a small proportion of genes were commonly influenced. The results presented here are more beneficial and can be considered as a complement to the previously reported studies; (2) The treatment conditions were varied among the published articles. Previous studies applied one-step cold or drought treatment, e.g., temperature directly drop from 28 to 7 °C [26] for cold and air withering- [24] or PEG6000- [30] simulated dehydration stress for drought. Differently, our study adopted a much more natural method of stress treatments: for cold, 5 days of 14 °C was added to trigger the potential chilling acclimation before 4 °C treatment; for drought, watering was stopped in rain-shed. Thus, the transcriptional changes of our study might be much closer to what happens to cassava in the field, and the cold and drought DE genes obtained here might be more suited for the genetic improvement of cold and drought tolerance in cassava.

Although they are of different characteristics, similar conclusions were observed among these studies. For examples, Utsumi et al. [24] and Fu et al. [30] reported that genes related to the signaling pathways of ABA, which is a well-characterized hormone that plays important roles in expression regulation of abiotic stresses in plants, were involved in the drought stress response in cassava. Moreover, this conclusion was confirmed in our present study, supporting the proposal that cassava has similar mechanisms for drought stress response as other plants [24]. An et al. [26] reported that the contents of two stress-responsive metabolites, malondialdehyde and proline, were significantly changed in response to cold stress in cassava. Similar phenotypes were observed when cassava was suffered to drought stress [30]. Correspondingly, the expression levels of ROS-scavenging genes (including dismutase, peroxidase, and catalase) were significantly altered in cold and drought stress conditions [29,30]. Similar conclusions were observed in our study, although the expression patterns of a few genes (e.g., *APX1*) were different when compared with previous studies [30], which might be due to their different treatment conditions. It has been demonstrated that the expression levels of genes related to photosynthesis were significantly inhibited by either cold or drought stress [26,30,31]. Interestingly, our study confirmed this result and further demonstrated that photosynthesis metabolism was greatly influenced by tetrapyrrole biosynthesis through the chlorophyll branch, specifically under cold condition.

In conclusion, our work provided a global overview of transcriptional changes of genes in response to both drought and cold stresses. These findings will improve our understanding of the specific and common responses to cold and drought in cassava, and can be considered as a useful complement to the previous studies which were performed only in cold or drought stress.

## 4. Materials and Methods

### 4.1. Plant Materials and Treatments

Stem segments with three nodes were extracted from 10-month-old cassava cv. SC124 plants, and inclined in 3 L pots filled with barren red soil/vermiculite (1:1, *v/v*) fertilized with Hoagland’s solution [57], to propagate and generate uniform cassava seedlings for experiments. The soil was supplied with 300 mL of normal-strength Hoagland’s solution once a week. The plants grew in natural conditions (light/dark of 13/11 h and 27–33/22–30 °C) for 60 days, and then uniform seedlings were tagged and subjected to cold and drought treatments.

For cold treatment, plants were transferred to an illuminated incubator at 24 °C for 2 days for a homogenous start, and temperature was then decreased from 24 to 14 °C at the rate of −2 °C/h to exert a moderate chilling stress. Temperature was then held constant at 14 °C for 5 days for chilling acclimation. After 5 days of chilling acclimation and growth at 14 °C, plants were watered once again with Hoagland’s solution, and cooled further to 4 °C at a rate of −2 °C/h, and cultivated at constant 4 °C for 5 days. Then, folded leaves (FL, the first three folded leaves), fully expanded leaves (FEL, three most newly fully expanded leaves), and fibrous roots (RT) were sampled from three individual plants at 6, 24, and 48 h, respectively. In parallel, plants grown under normal condition of 24 °C at the three corresponding time points served as controls.

For drought treatment, watering of uniform plants was stopped for 5 days to stimulate the acclimation effects. At 6 days, the plants were watered once more with Hoagland’s solution, and then watering was stopped for another 10 days (i.e., the second phase) for the drought treatment. After 4, 6, and 10 days of the second phase of drought treatment, FL, FEL, and RT were harvested respectively from three individual plants. At the corresponding times, the same tissues of control plants (maintained at 90% full field water content by supplying the amount of water determined from weight loss) were also sampled. All samples were immediately frozen in liquid nitrogen and stored at −80 °C.

### 4.2. RNA-Seq Library Preparation and Sequencing

Total RNA was isolated separately for each sample as previously described. The integrity and quality of the total RNA were examined using a Nanodrop ND-1000 spectrophotometer (Thermo Scientific Inc., Waltham, MA, USA) and an Agilent 2100 Bioanalyzer (Agilent, Santa, CA, USA). For each time point of the treatment, equal amounts of RNA of the same tissue from three individual plants were pooled for expression profiling. In total, 24 RNA-seq libraries were separately constructed and sequenced at Beijing Genomics Institute (Shenzhen, China) based on the Illumina GAII platform.

### 4.3. Data Analysis

As previously described [58,59], adapters were removed from raw sequence reads using FASTX-toolkit version 0.0.13 (Available online: http://hannonlab.cshl.edu/fastx_toolkit/). Sequence quality was examined using FastQC (Available online: http://www.bioinformatics.babraham.ac.uk/projects/fastqc/), and reads with low quality were removed using parameter q20p80, which indicates 80% of bases had an accuracy of >99%. Reads were then mapped to the cassava genome (version 4.1) obtained from the Phytozome website (Available online: ftp://ftp.jgi-psf.org/pub/compgen/phytozome/v9.0/Mesculenta/) using Tophat v2.0.10 (Available online: http://tophat.cbcb.umd.edu/) [60]. After trimmed mean of M-values normalization, raw read counts were used to calculate CPM (Counts Per Million mapped reads) for gene expression level. Expressed genes were arbitrarily defined as those with CPM > 10.

Differentially expressed (DE) genes were identified through two steps. (1) For each tissue (which included one control and three treatment time points) in either cold or drought stress, we used similar previously reported criteria [29] to determine whether a gene was a DE gene candidate. For example, given two samples of one treatment and one control, a gene was considered as a DE gene candidate if (a) the fold-change (FC) of CPM was >3 if the gene was expressed in two samples, or (b) the gene was not expressed (CPM < 10) in one sample but over-expressed (CPM > 30) in the other sample. (2) DE gene candidates that were significant in at least two of three time points during the first step were selected and finally considered as DE genes in response to stress in that tissue. This step could be considered as a complement to the lack of replicates, which used different time points instead.

To understand the gene interactions between cold and drought stresses, and among different tissues (e.g., FL, FEL, and RT), heatmaps were used to visualize the FC of gene expression, which were grouped by hierarchical clustering (with “complete” method according to the Euclidean distance) implemented in MeV [61]. The FCs were all set to 1 for non-DE genes. For gene functional category enrichment, cassava loci were functionally annotated and classified into hierarchical categories based on MapMan [62], and then significantly over-represented functional categories were identified based on Fisher’s exact test as previously reported [30,58].

### 4.4. Validation of Commonly and Specifically Expressed Genes Responding to Cold and Drought Treatments

To validate the results of RNA-seq, seven genes of interest, related to primary cell wall process, stress responsive transcriptional factors, and protein modification and degradation, were selected and confirmed by using qRT-PCR with the primers listed in Table S1. The amplification programs were performed on an ABI PRISM 7900HT real-time PCR system. The amplification conditions were 95 °C for 30 s, followed by 40 cycles of 95 °C for 5 s and 60 °C for 30 s. A thermal denaturing step that generated the melt curves was followed after the qRT-PCR cycles to verify the amplification specificity. Beta-actin was used as the endogenous control. Each sample was measured in triplicate, including its non-template control. The relative gene expression level across the samples was calculated by using the 2^–^^△△*C*t^ method.

## Supplementary Materials

Supplementary materials can be found at www.mdpi.com/1422-0067/18/12/2668/s1.
