# Supplementary material for: The Discrepant and Similar Responses of Genome-Wide Transcriptional Profiles between Drought and Cold Stresses in Cassava
Source: Int J Mol Sci. 2017 Dec 12;18(12):2668. doi: 10.3390/ijms18122668 (PMC5751270; doi:10.3390/ijms18122668)
Supplement: Supplementary file 1 [file ijms-18-02668-s001.zip › Supple_Tab_Fig/Supplementary.pdf]

The Discrepant and Similar Responses of Genome-Wide Transcriptional Profiles between Drought and Cold Stresses in Cassava

Changying Zeng †, Zehong Ding †, Fang Zhou, Yufei Zhou, Ruiju Yang, Zi Yang, Wenquan Wang and Ming Peng *

Institute of Tropical Bioscience and Biotechnology, Chinese Academy of Tropical Agricultural Sciences, Haikou 571101, Hainan, China; zengchangying@itbb.org.cn (C.Z.); dingzehong@itbb.org.cn (Z.D.); fzhou_413@aliyun.com (F.Z.); zyf20080825@163.com (Y.Z.); wxxnyrj@126.com (R.Y.); yangzi8866@126.com (Z.Y.); wangwenquan@itbb.org.cn (W.W.)

* Correspondence: pengming@itbb.org.cn; Tel.:+86-898-6689-0981

† These authors contribute equally in this work.

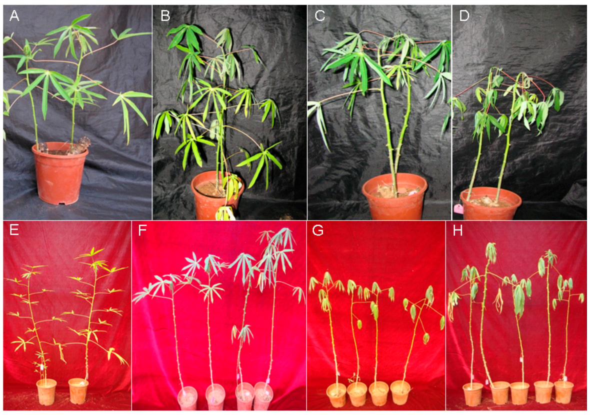

Figure S1 Phenotypes of cassava under cold and drought stresses. (A-D) represent plants grown under cold stress for 0 h (control), 6 h, 24 h, and 48 h, respectively. (E-H) represent plants grown under drought stress for 0 d (control), 4 d, 6 d, and 10 d, respectively.

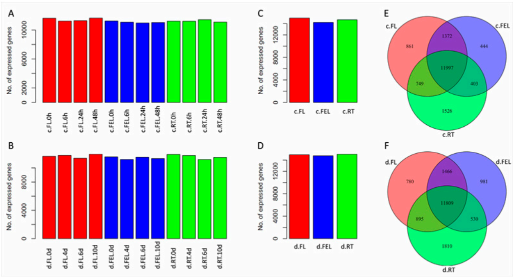

Figure S2 Number of expressed genes and their comparisons among different tissues in cassava. Expressed genes identified in 12 samples (A & B), three tissues (C & D) and their Venn diagrams (E & F) in cold and drought stresses, respectively.

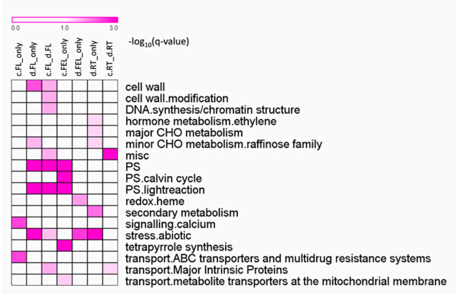

Figure S3 Functional category enrichment for the DE genes compared between cold and drought treatments within the same tissue.

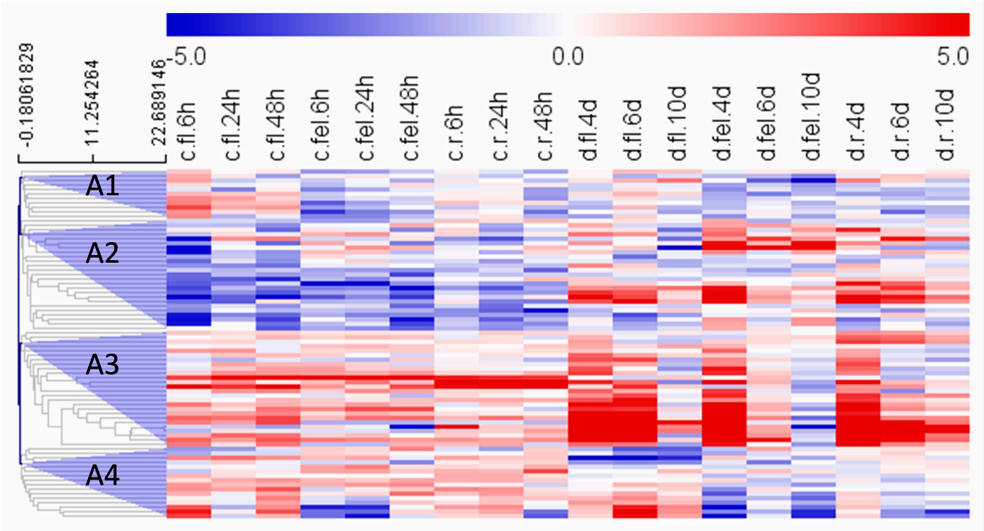

Figure S4 Heatmap of abiotic stress related genes. In total 4 groups of genes were identified. Blue represents down-regulation, and red represents up-regulation. Samples were named by combination of stresses ('c' for cold, and 'd' for drought), tissues, and time points as indicated in Figure 2.
